# *YWHAG* Mutations Cause Childhood Myoclonic Epilepsy and Febrile Seizures: Molecular Sub-regional Effect and Mechanism

**DOI:** 10.3389/fgene.2021.632466

**Published:** 2021-03-09

**Authors:** Xing-Guang Ye, Zhi-Gang Liu, Jie Wang, Jie-Min Dai, Pei-Xiu Qiao, Ping-Ming Gao, Wei-Ping Liao

**Affiliations:** ^1^Department of Pediatrics, Affiliated Foshan Maternity & Child Healthcare Hospital, Southern Medical University, Foshan, China; ^2^Zhujiang Hospital, Southern Medical University, Guangzhou, China; ^3^Department of Neurology, Institute of Neuroscience, The Second Affiliated Hospital of Guangzhou Medical University, Guangzhou, China; ^4^Key Laboratory of Neurogenetics and Channelopathies of Guangdong Province and the Ministry of Education of China, Guangzhou, China

**Keywords:** *YWHAG* gene, 14-3-3γ, myoclonic epilepsy, febrile seizures, molecular sub-regional effect

## Abstract

*YWHAG*, which encodes an adapter protein 14-3-3γ, is highly expressed in the brain and regulates a diverse range of cell signaling pathways. Previously, eight *YWHAG* mutations have been identified in patients with epileptic encephalopathy (EE). In this study, using trios-based whole exome sequencing, we identified two novel *YWHAG* mutations in two unrelated families with childhood myoclonic epilepsy and/or febrile seizures (FS). The identified mutations included a heterozygous truncating mutation (c.124C>T/p.Arg42Ter) and a *de novo* missense mutation (c.373A>G/p.Lys125Glu). The two probands experienced daily myoclonic seizures that were recorded with ictal generalized polyspike-slow waves, but became seizure-free with simple valproate treatment. The other affected individuals presented FS. The truncating mutation was identified in the family with six individuals of mild phenotype, suggesting that *YWHAG* mutations of haploinsufficiency are relatively less pathogenic. Analysis on all missense mutations showed that nine mutations were located within 14-3-3γ binding groove and another mutation was located at residues critical for dimerization, indicating a molecular sub-regional effect. Mutation Arg132Cys, which was identified recurrently in five patients with EE, would have the strongest influence on binding affinity. 14-3-3γ dimers supports target proteins activity. Thus, a heterozygous missense mutation would lead to majority dimers being mutants; whereas a heterozygous truncating mutation would lead to only decreasing the number of wild-type dimer, being one of the explanations for phenotypical variation. This study suggests that *YWHAG* is potentially a candidate pathogenic gene of childhood myoclonic epilepsy and FS. The spectrum of epilepsy caused by *YWHAG* mutations potentially range from mild myoclonic epilepsy and FS to severe EE.

## Introduction

*YWHAG* gene (OMIM^*^ 605356) resides on 7q11.23 and encodes tyrosine 3-monooxygenase/tryptophan 5-monooxygenase activation protein gamma (14-3-3γ). This protein regulates a diverse range of cell signaling pathways by forming protein–protein complexes with signaling proteins within specific sequence motifs (Aghazadeh and Papadopoulos, 2016). The 14-3-3γ protein is highly conserved and expressed in the mammalian brain and plays a critical role in neuronal migration and morphological defects in the developing cortex (Mizuno et al., 2007; Cornell et al., 2016; Wachi et al., 2016). *YWHAG* mutations have been demonstrated to be associated with epileptic encephalopathy (Consortium et al., 2013; Guella et al., 2017; Kanani et al., 2020), and occasionally identified in patients with developmental disorders and autism (De Rubeis et al., 2014; McRae et al., 2017). It is unknown whether *YWHAG* mutations are associated with other phenotypes of epilepsy.

Febrile seizures (FS) are the most common convulsions in childhood. Some of the children with FS also have afebrile seizures or develop to epilepsy later. The genes associated with FS/FS-related epilepsy include *ADGRV1, CPA6, DYRK1A, FEB2, FEB5, FEB6, FEB7, FEB9, FEB10, FGF13, GABRB3, GABRD, GABRG2, GEFSP4, GEFSP6, GEFSP8, HCN1, NPRL3, SCN1A, SCN1B, SCN2A, SCN9A*, and *STX1B* (OMIM, https://www.omim.org/). Here, we performed trios-based whole exome sequencing approach in a cohort of patients with FS plus. Two *YWHAG* mutations were identified in two unrelated families with childhood myoclonic epilepsy and FS, including a familial case with six individuals affected and a sporadic case with *de novo* mutation. We systematically reviewed the *YWHAG* variants and analyzed the potential molecular consequences of the mutations.

## Materials and Methods

### Subjects

The patients were from the department of pediatrics, Foshan Maternity & Child Healthcare Hospital from July 2018 to April 2020.

Clinical information of the affected individuals were collected, including detailed information on seizures, general and neurological examination results, family history, and response to antiepileptic drugs (AEDs). Magnetic resonance imaging (MRI) scans were performed to detect any brain structure abnormalities. Long-term Video- electroencephalography (EEG) monitoring records that included hyperventilation, intermittent photic stimulation, open-close eyes test, and sleeping recording were obtained. Epileptic seizures and epilepsies were diagnosed according to the criteria of the Commission on Classification and Terminology of the ILAE (1981, 1989, 2001, 2010, and 2017). FS was defined as a seizure occurring in childhood after 1 month of age associated with a febrile illness not caused by an infection of the central nervous system, without previous neonatal seizures or a previous unprovoked seizure, and not meeting the criteria for other acute symptomatic seizures. FS plus was used to denote the individuals with FS extending outside the age range of 3 months to 6 years, or with any forms of afebrile seizures, including generalized seizure like myoclonic seizures. A total of 36 subjects with FS plus were recruited in this study. The patients and their parents (trios), and the other familial members available were screened for genetic variants by whole exome sequencing. In cases with more than one individual affected, all familial members available were sequenced. Before whole exome sequencing, no other genetic analysis, such as gene panel for epilepsy and CGH-array, were performed.

This study was approved by the ethics committee of Foshan Maternity & Child Healthcare Hospital, and all participants undergoing testing consented to their data being used for research.

### Whole Exome Sequencing

Whole blood samples were collected from the available subjects and used for linkage and segregation analysis. Qiagen Flexi Gene DNA kit (Qiagen, Hilden, Germany) was used to isolate genomic DNA for exome enrichment. Whole exome sequencing was performed with NextSeq500 sequencing instruments (Illumina, San Diego, California). Sequence alignment and variant calling were performed according to standard procedures as previously described (Wang et al., 2018). All candidate pathogenic variants were validated by Sanger sequencing. The variants with nucleotide and amino acid were numbered according to *YWHAG* reference transcript NM_012479.3 and reference protein NP_036611.2.

### Molecular Structural Analysis

Protein modeling was performed to predict the effects of missense variants on molecular structure by using SWISS-MODEL (https://swissmodel.expasy.org/), based on the updated template of 4J6S.pdb (chain A) (http://www.rcsb.org) (Shen et al., 2018). PyMOL 1.7 was used for three-dimensional protein structure visualization and analysis. mCSM-PPI2 was used to calculate value of affinity change (ΔΔG^Affinity^ = kcal/mol) (Rodrigues et al., 2019), and predicted the effects of variants on protein-protein interactions. Variants were discriminated into two classes: decreasing affinity (ΔΔG^Affinity^ <0 kcal/mol) and increasing affinity (ΔΔG^Affinity^ > 0 kcal/mol).

## Results

### Identification of Novel *YWHAG* Mutations

**Figure 1:**
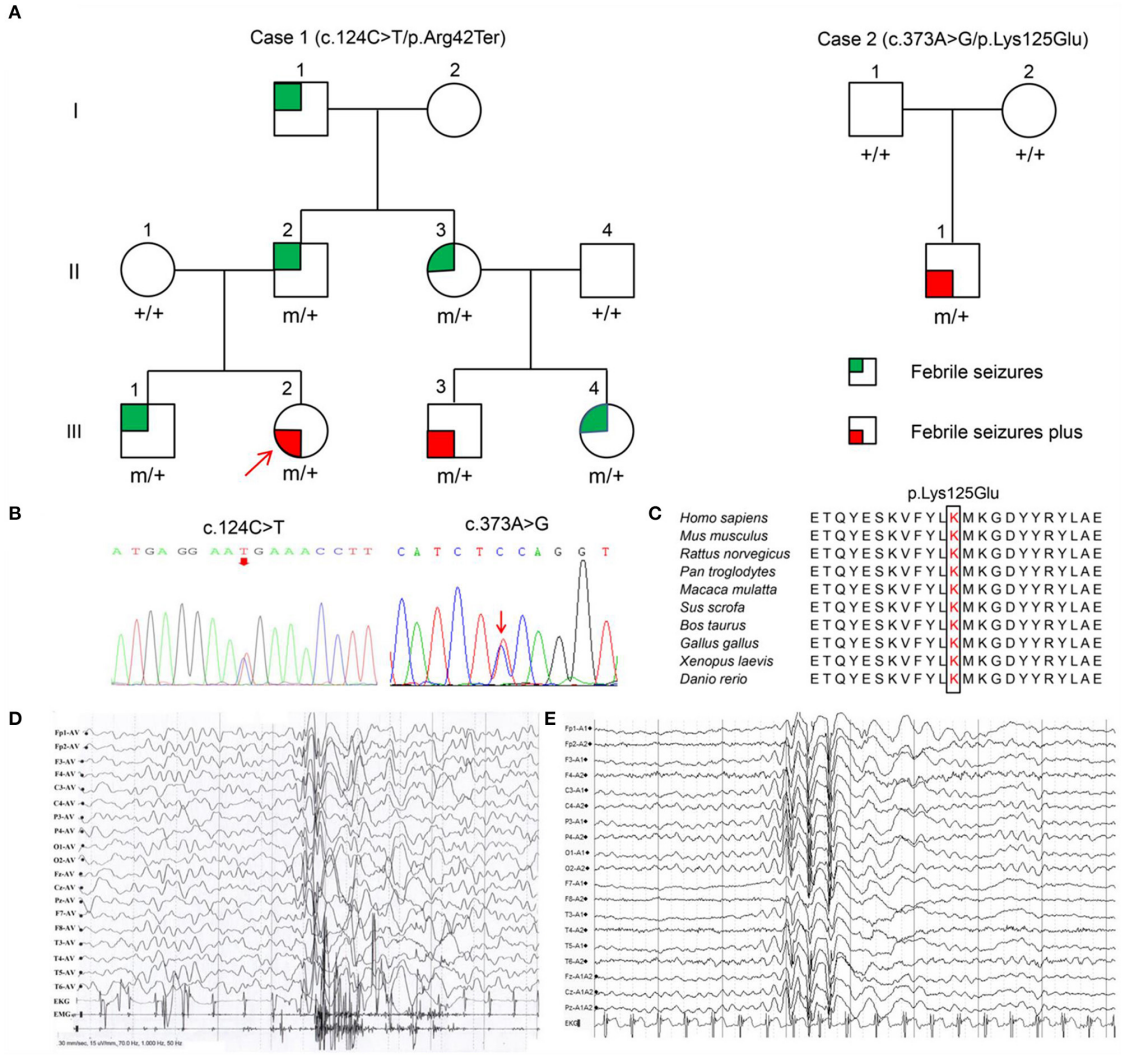
Pedigrees, *YWHAG* mutations and EEG changes in the proband. **(A)** Pedigrees of the families with *YWHAG* mutations. Individuals with mutation are indicated by m/+, and those negative for mutation are indicated by +/+. **(B)** Sequencing chromatograms of c.124C>T and c.373A>G. **(C)** The amino acid sequence alignment of 14-3-3γ showed that the residue Lys125 was highly conserved in various species. **(D)** Ictal EEG changes in the proband with mutation Arg42Ter showed myoclonic seizures with generalized irregular polyspike-and-slow waves (obtained at the age of 1 year). **(E)** Interictal EEG changes in the proband with mutation Lys125Glu showed generalized spike-and-slow waves (obtained at the age of 2 years).

Among the 36 patients with FS plus, two *YWHAG* mutations were identified in two unrelated cases (Figure 1A, Table 1). The two probands also presented myoclonic seizures. A heterozygous truncating mutation (c.124C>T/p.Arg42Ter) (Figure 1B) was identified in a family, in which the mutation was fully co-segregated with the phenotypes. Mutation Arg42Ter introduces a premature stop codon, potentially resulting in a truncated 14-3-3γ protein and leading to functional haploinsufficiency. A *de novo* missense mutation (c.373A>G/p.Lys125Glu) (Figure 1B) was identified in a case with childhood myoclonic epilepsy and FS. This mutation affected an amino acid residue that is highly conserved in various species (Figure 1C) and was suggested to be deleterious by 18 out of 21 prediction tools (Supplementary Table 1). The two mutations were not present in the general population on the databases of gnomAD and 1000 Genomes Project, and evaluated as pathogenic and likely pathogenic by the standards and guidelines of the American College of Medical Genetics and Genomics, respectively (Table 2).

**Table 1:** Clinical feature of patients with *YWHAG* mutations.

**Subjects**	**Gender**	**Age**	**Seizure onset**	**Seizure course**	**EEG**	**MRI**	**Effective AEDs**	**Diagnosis**
**Case 1 (c.124C>T/p.Arg42Ter)**								
II-2	Male	40 yr	<1 yr	GTCS (FS), 1–2 times/yr for 4 yr	NA	NA	NA	FS
II-3	Female	38 yr	<1 yr	GTCS (FS), 1–2 times/yr for 4 yr	NA	NA	NA	FS
III-1	Male	14 yr	2 yr	GTCS (FS), 1–2 times/yr for 5 yr	NA	NA	NA	FS
III-2	Female	3 yr	7 mo	GTCS (FS and aFS), 5 times/yr for 1 yr; myoclonic seizures, 10 times/d for 1 mo	Ictal: myoclonic seizures with generalized irregular polyspike-slow waves; interictal: no discharge	Normal	VPA	FS+, myoclonic epilepsy
III-3	Male	4 yr	8 mo	GTCS (FS and aFS), 5 times/yr for 1 yr	NA	Normal	VPA	FS+
III-4	Female	8 yr	2 yr	GTCS (FS), 1–2 times/yr for 3yr	NA	NA	NA	FS
**Case 2 (c.373A>G/p.Lys125Glu)**								
B: II-1	Male	3 yr	19 mo	GTCS (FS and aFS), 7 times/yr for 1 yr; myoclonic seizures, 3 times/d for 1 mo	Generalized spike-slow waves	Normal	VPA	FS+, myoclonic epilepsy

*AEDs, antiepileptic drugs; aFS, afebrile seizure; EEG, electroencephalogram; FS, febrile seizures; FS+, febrile seizures plus; GTCS, generalized tonic-clonic seizures; MRI, magnetic resonance imaging; NA, not available; VPA, valproate; yr, year; mo, month*.

**Table 2:** Genetic feature of *YWHAG* mutations.

**cDNA change (NM_012479.3)**	**Protein change (NP_036611.2)**	**Phenotype**	**MAF**	**Inheritance**	**Damaging prediction^‡^**	**ΔΔG^**Affinity**^ (kcal/mol)^§^**	**PIS**	**ACMG (scoring)**
c.124C>T^†^	p.Arg42Ter	FS+, myoclonic seizures	0	Family	–	–	–	P (PVS1 + PM2 + PP1)
c.373A>G^†^	p.Lys125Glu	FS+, myoclonic seizures	0	*de novo*	18	−0.635	No	LP (PS2 + PM2 + PP3)
c.44A>C	p.Glu15Ala	Epileptic encephalopathy	0	*de novo*	15	−0.620	Yes^a^	LP (PS2 + PM2 + PP3)
c.169C>G	p.Arg57Gly	Epileptic encephalopathy	0	*de novo*	19	−0.517	Yes^b^	LP (PS2 + PM2 + PP3)
c.169C>T	p.Arg57Cys	Epileptic encephalopathy	0	*de novo*	19	−0.360	Yes^b^	LP (PS2 + PM2 + PP3)
c.387C>G	p.Asp129Glu	Epileptic encephalopathy	0	*de novo*	19	−0.969	Yes^b^	LP (PS2 + PM2 + PP3)
c.394C>T	p.Arg132Cys	Epileptic encephalopathy	0	*de novo*	18	−1.288	Yes^b^	LP (PS2 + PM2 + PP3)
c.398A>C	p.Tyr133Ser	Developmental delay, epileptic encephalopathy	0	*de novo*	19	−0.611	Yes^b^	LP (PS2 + PM2 + PP3)
c.529C>A	p.Leu177Ile	Epileptic encephalopathy	0	*de novo*	18	−1.193	Yes^c^	LP (PS2 + PM2 + PP3)
c.532A>G	p.Asn178Asp	Epileptic encephalopathy	0	*de novo*	18	−0.010	Yes^b^	LP (PS2 + PM2 + PP3)
c.148A>C	p.Lys50Gln	Autism spectrum disorder	0	*de novo*	18	−0.534	No	LP (PS2 + PM2 + PP3)

*ACMG, American College of Medical Genetics and Genomics (P, pathogenic; LP, likely pathogenic); FS+, febrile seizure plus; MAF, minor allele frequency from gnomAD; PIS, primary interaction sites; PVS1, Predicted null variant in a gene where loss-of-function is a known mechanism of disease; PS2, De novo in a patient with the disease and no family history; PM1, Located in a mutational hot spot and/or critical and well-established functional domain (e.g., active site of an enzyme) without benign variation; PM2, Absent in population databases; PP1, Cosegregation with disease in multiple affected family members in a gene definitively known to cause the disease; PP3, Multiple lines of computational evidence support a deleterious effect on the gene/gene product*.

† *Variant identified in this study*.

‡ *Variant predicted to be damaging out of 21 prediction tools*.

§ *Protein-protein affinity change upon variant indicated by free energy change*.

a *A crucial site involved in 14-3-3γ dimerization*.

b *A part of conserved triads (Arg132-Arg57-Tyr133 and Asp129-Asn178-Asn229) for fixing the orientation of the phosphopeptides*.

c *A part of conserved hydrophobic patch (Leu-177-Leu-221-Ile-222) complements the hydrophobic character of the peptide*.

### Clinical Information

The clinical information of patients with *YWHAG* mutations was summarized in Table 1. The truncating mutation Arg42Ter was identified in a familial case with six individuals affected. The proband was 3-year-old girl who experienced simple FS at the age of 7 months, occurred around 5 times per year. Subsequently, she experienced daily myoclonic seizures since 1 year old. EEGs recorded multiple myoclonic seizures with generalized irregular polyspike-and-slow waves (Figure 1D). She was born to unrelated parents after an uneventful pregnancy. Her MRI was normal. She got seizure-free with valproate on a dose of 25 mg/kg/day. Her intelligent and development were normal. Five other members in the family were affected by FS or FS plus (afebrile generalized tonic-clonic seizures) (Figure 1A, Table 1) that were co-segregated with the mutation. They all had normal intelligent and physical developments, and had got seizure-free without medication.

The missense mutation Lys125Glu was identified in a 3 years old boy who experienced a simple FS at 19 months old. The FS occurred 7 times in the following year. He started to have myoclonic seizures since 2 years old. EEGs showed generalized spike-and-slow waves (Figure 1E). His MRI was normal. He was born to unrelated parents after an uneventful pregnancy. He got seizure-free with valproate on a dose of 25 mg/kg/day. He had normal intelligent and motor development.

### Effect of Missense on Molecular Structure

Previously, eight *de novo YWHAG* mutations have been identified in patients with epileptic encephalopathy (EE), including Glu15Ala, Arg57Gly, Arg57Cys, Asp129Glu, Tyr133Ser, Leu177Ile, Asn178Asp in one patient each, and Arg132Cys recurrently in five unrelated cases (Kanani et al., 2020). Tyr133Ser has also been identified in a patient with neurodevelopmental disorder (McRae et al., 2017). Additionally, a *de novo* mutation Lys50Gln was identified in one patient with autism (De Rubeis et al., 2014). To explore the possible association between *YWHAG* mutations and diseases, we analyzed the potential molecular consequences of the mutations.

**Figure 2:**
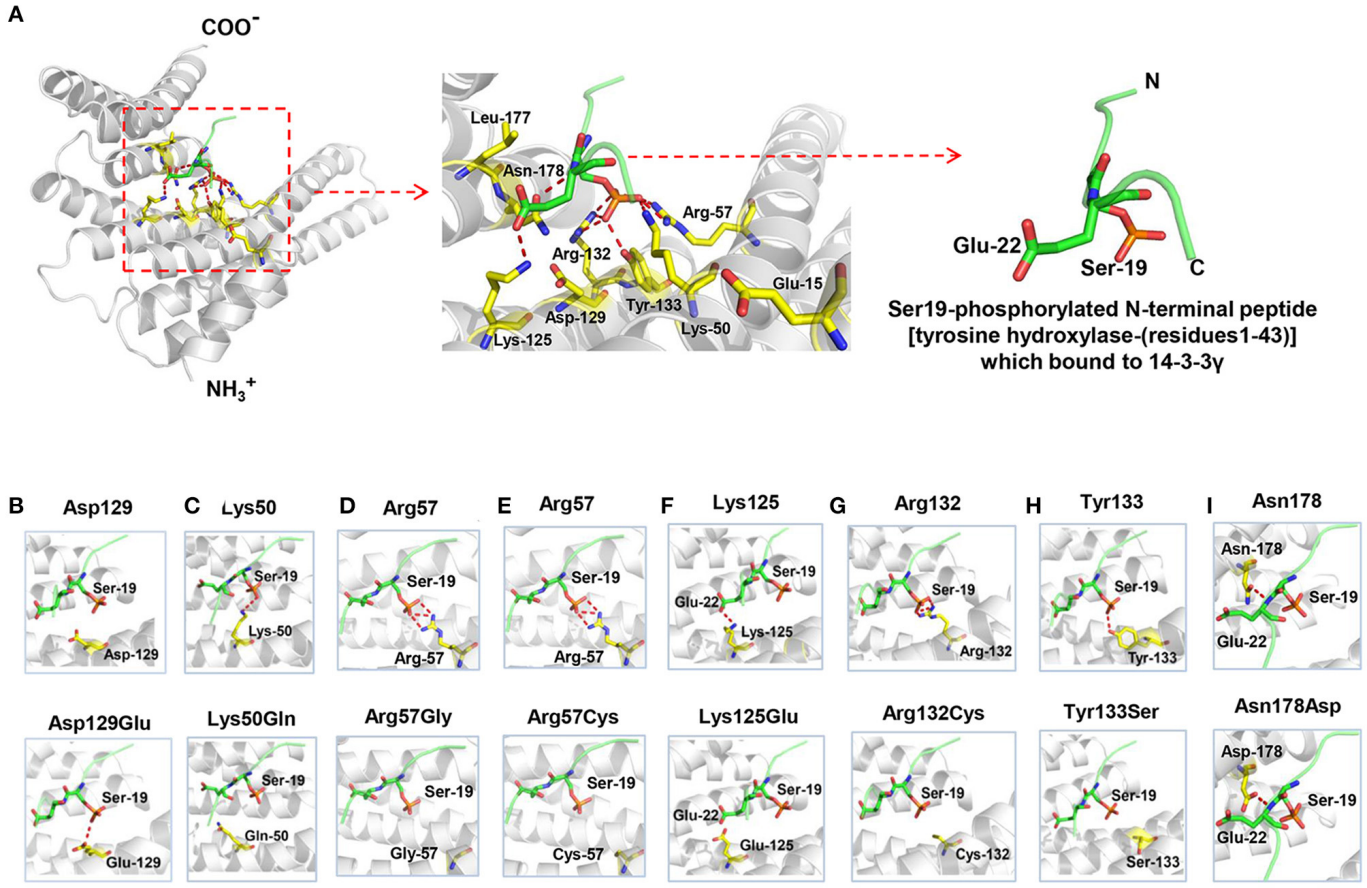
Crystal structure of 14-3-3γ (PDB: 4J6S) and hydrogen bond changes of the mutants. **(A)** Left: monomeric 14-3-3γ is shown as gray ribbons, and the phosphopeptide ligand is shown as a green stick. Middle: close-up view of the binding groove and the side chains of the residues where mutations occurred. Right: Ser19-phosphorylated N-terminal peptide [tyrosine hydroxylase-(residues1-43)], which bound to 14-3-3γ. **(B)** Substitution of aspartate with glutamate at residue 129 (Asp129Glu) led to a new hydrogen bond with phosphopeptide ligand. **(C)** Substitution of lysine with glutamate at residue 50 (Lys50Gln) destroyed the original hydrogen bond with phosphopeptide ligand. **(D)** Substitution of arginine with glycine at residue 57 (Arg57Gly) destroyed the original hydrogen bond with phosphopeptide ligand. **(E)** Substitution of arginine with cysteine at residue 57 (Arg57Cys) destroyed the original hydrogen bond with phosphopeptide ligand. **(F)** Substitution of lysine with glutamate at residue 125 (Lys125Glu) destroyed the original hydrogen bond with phosphopeptide ligand. **(G)** Substitution of arginine with cysteine at residue 132 (Arg132Cys) destroyed the three original hydrogen bonds with phosphopeptide ligand. **(H)** Substitution of tyrosine with serine at residue 133 (Tyr133Ser) destroyed the original hydrogen bond with phosphopeptide ligand. **(I)** Substitution of asparagine with aspartate at residue 178 (Asn178Asp) formed a similar hydrogen bond with phosphopeptide ligand.

The 14-3-3γ protein is composed of a dimerization region located at the amino-terminus and a target-protein-binding region (Fu et al., 2000). Among the ten missense mutations, seven mutations were related to the binding sites of Ser19-phosphorylated peptide of tyrosine hydroxylase (THp, Figure 2A) (Tzivion et al., 2001; Skjevik et al., 2014). Mutation Lys50Gln, Arg57Gly, Arg57Cys, Arg132Cys, Tyr133Ser, Asn178Asp, and Lys125Glu that identified in the present study, were located at residues that had direct hydrogen bonds with THp. Asp129 is a conserved residue involved in fixing the orientation of the phosphorylated peptides (Yang et al., 2006). It did not have direct hydrogen bonds with THp, but become to have hydrogen bond connection with THp when Asp129Glu occurred (Figure 2B). Leu177 is close to, but not a direct binding site of THp. However, it is part of a conserved triad of two leucine and a isoleucine (Leu-177, Leu-221, and Ile-222) that forms a hydrophobic patch within the 14-3-3 binding groove that complements the hydrophobic character of the peptide on the C-terminal side of the phosphoserine (Yang et al., 2006). Glu15 is a crucial residue involved in 14-3-3γ dimerization that is required for binding to certain target proteins (Zhou et al., 2003; Valente et al., 2012). Figure 2A shows all missense mutations were located within or close to 14-3-3γ binding groove, in regions potentially involved in target protein binding. Their relationships with primary interaction sites (Tzivion et al., 2001; Yang et al., 2006; Skjevik et al., 2014) were summarized in Table 2. The EE associated *YWHAG* mutations were all located at the primary interaction sites, whereas the mutation Lys125Glu did not.

Protein modeling showed that Lys50, Arg57, Arg132, and Tyr133 formed hydrogen-bonds with Ser19 of THp; Lys125 and Asn178 formed a hydrogen-bond with Glu22 of THp. The mutations Lys50Gln, Arg57Gly, Arg57Cys, Lys125Glu, Arg132Cys, and Tyr133Ser destroyed these hydrogen bonds (Figures 2C–H, respectively). Substitution of asparagine with aspartate at residue 178 (Asn178Asp) formed a new hydrogen bond with phosphopeptide ligand (Figure 2I).

The effects of mutations on protein-protein interaction binding affinity were further analyzed using mCSM-PPI2. All ten mutations were predicted to decrease 14-3-3γ binding affinity (Table 2). Mutation Arg132Cys identified recurrently in five EE patients would have relatively stronger influence on 14-3-3γ binding affinity (ΔΔG^Affinity^ = −1.288 kcal/mol) than the others, suggesting that change of protein-protein interaction was potentially associated with the phenotype variations.

## Discussion

The 14-3-3γ is a small acidic protein that exists primarily as homo- and heterodimers (Fu et al., 2000). It is highly conserved and expressed in the mammalian brain and has been found to interact with critical regulatory proteins controlling a wide array of signaling pathways (Aghazadeh and Papadopoulos, 2016). Previously, eight *YWHAG* mutations Glu15Ala, Arg57Gly, Arg57Cys, Asp129Glu, Arg132Cys, Tyr133Ser, Leu177Ile, and Asn178Asp were identified in twelve patients with EE (Guella et al., 2017; Kanani et al., 2020). Mutations Arg132Cys and Tyr133Ser were recurrently identified in EE patients with variable seizure type and onset age, and responses to antiepileptic therapy. All individuals had developmental delay with intellectual disability, and one patient was also diagnosed with autism spectrum disorder. In this study, two novel *YWHAG* mutations were identified in two unrelated families with childhood myoclonic epilepsy and FS, including a familial case with six individuals affected and a case with *de novo* mutation. The two probands also experienced daily myoclonic seizures (Table 1). EEGs recorded multiple myoclonic seizures with generalized polyspike-and-slow waves (Figures 1D,E). They have got seizure-free with simple valproate treatment. The two probands could be diagnosed as benign myoclonic epilepsy in infancy (Darra et al., 2006), which is overlapped in onset age with early childhood myoclonic epilepsy (Yang et al., 2017). The others with simple FS have got seizure-free without medication. All patients from this study have normal intelligent and motor development. These evidences indicate that *YWHAG* mutations are potentially associated with mild phenotypes like myoclonic epilepsy and FS with favorable outcomes. Previously, myoclonic seizures and FS have also been observed in severe EE cases with mutations Glu15Ala and Arg132Cys, consistent with the observation in this study and supporting the association between *YWHAG* mutations and myoclonic epilepsy/FS. Therefore, the spectrum of epilepsy caused by *YWHAG* mutations potentially range from mild myoclonic epilepsy and FS to severe EE.

The mutation Arg42Ter was identified in a large pedigree, which potentially resulted in a truncated 14-3-3γ protein and led to functional haploinsufficiency. Previous studies showed that 14-3-3γ deficiency in Zebrafish leads to reduced brain size and delayed brain development (Komoike et al., 2010). Ablation of 14-3-3γ in mice results in delayed neuronal migration of pyramidal neurons in the cerebral cortex (Mizuno et al., 2007). These data suggest that loss-of-function of *YWHAG* is potentially pathogenic. Given the comparatively mild phenotype produced by the truncating mutation, it is suggested that *YWHAG* mutations of haploinsufficiency (pure loss of function) are relatively less pathogenic.

**Figure 3:**
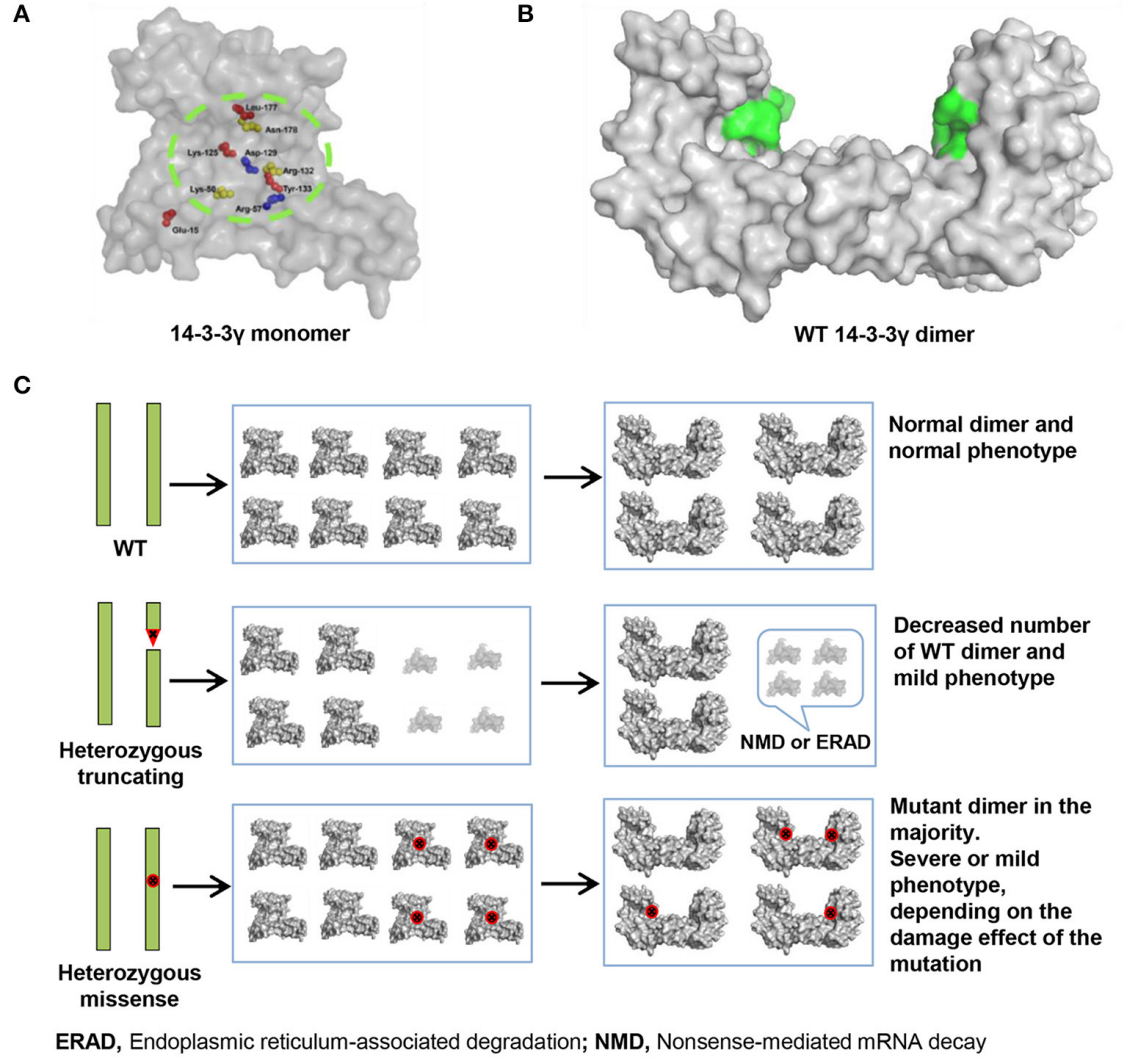
14-3-3γ dimer forming and its association with phenotypical variation. **(A)** A front view of 14-3-3γ monomer shows the location of the missense mutations. **(B)** A lateral view of 14-3-3γ dimer. The peptide-binding grooves are colored as green surface. **(C)** Difference in the pathogenic mechanism between heterozygous truncating mutation and heterozygous missense mutation. A heterozygous truncating mutation would lead to only decreasing the number of wild-type dimer, whereas a heterozygous missense mutation would lead to majority dimers being mutants. The binding groove with missense mutation is colored red.

The main feature of 14-3-3γ protein is its ability to bind to target proteins, thus to cause changes in their activity, modification, and intracellular localization (Bridges and Moorhead, 2004; Jin et al., 2004; Obsilova et al., 2008; Lundby et al., 2012). So far, ten missense mutations have been reported, including eight mutations (Glu15Ala, Arg57Gly, Arg57Cys, Asp129Glu, Arg132Cys, Tyr133Ser, Leu177Ile, and Asn178Asp) associated with EE (Kanani et al., 2020), one mutation (Lys125Glu) associated with childhood myoclonic epilepsy and FS (the present study), and one mutation (Lys50Gln) associated with autism (De Rubeis et al., 2014). Interestingly, we found that nine mutation residues except Glu15 are located within 14-3-3γ binding groove for phosphorylated peptide ligand (Figure 3A) (Tzivion et al., 2001; Yang et al., 2006; Skjevik et al., 2014). Especially, Arg132-Arg57-Tyr133 is a highly conserved triad within 14-3-3γ binding groove for fixing the orientation of the phosphopeptides, Asp129 is a conserved residue involved in fixing the orientation of the phosphorylated peptides (together with Asn178), and Leu177 is part of a conserved hydrophobic patch within the 14-3-3γ binding groove that complements the hydrophobic character of the peptide on the C-terminal side of the phosphoserine (Yang et al., 2006). These residues are primary interaction sites responsible for 14-3-3γ-phosphopeptide interactions. Substitution at these residues destroyed the hydrogen bonds with peptide ligand and potentially affected ligand backbone binding (Figure 2, Table 2). Glu15 is a crucial residue involved in 14-3-3γ dimerization (Zhou et al., 2003; Valente et al., 2012), and Glu15Ala potentially impaired 14-3-3γ dimerization, which is required for binding to target proteins (Tzivion et al., 2001). All *YWHAG* missense mutations were located in regions for target protein binding (Figure 3A). The EE associated *YWHAG* mutations were all located at the primary interaction sites. In contrast, the mutation Lys125Glu, which was identified in the patient with mild myoclonic epilepsy, was located outside the critical sites (Table 2). This may potentially be one of the explanations for phenotypic variation. Our previous study suggests that the molecular sub-regional location was a critical factor to determine the damaging effect and pathogenicity of the mutations (Tang et al., 2019). The present study suggested that the impaired molecular sub-regions involved in target protein binding for 14-3-3γ play a potential role in the underlying mechanism of pathogenesis and phenotypic variations.

Analysis on the effects of *YWHAG* missense mutations on protein–protein interaction binding affinity showed that mutations Arg132Cys, which were identified in five EE patients, would have relatively stronger influence on 14-3-3γ binding affinity than the others (Table 2), potentially being one of explanations for the severe phenotype. Previous study revealed that only the dimeric form of 14-3-3γ supports target proteins activity (Figure 3B); and mutant 14-3-3γ with missense mutations would produce abnormal 14-3-3γ dimers, which consist of a wild-type 14-3-3γ monomer and a mutant monomer with impaired ability of binding to target proteins (Figure 3C) (Muslin and Xing, 2000). Thus, a heterozygous missense mutation would lead to majority dimers being mutants (Figure 3C), and the phenotype severity potentially depends on the damaging effect of the missense mutation. In contrast, a heterozygous truncating mutation would lead to only decreasing the number of wild-type dimer (Figure 3C), due to the inability of mutants in forming dimers, explaining the mild phenotype associated with heterozygous truncating mutations.

14-3-3γ proteins have a variety of downstream proteins. It is unknown which of the downstream proteins are involved in the pathogenesis of epilepsy. TH protein is among the first binding partners associated with several neurological diseases (Ichimura et al., 1988; Dunkley et al., 2004; Daubner et al., 2011; Aumann and Horne, 2012). It catalyzes the rate-limiting step in the synthesis of catecholamine neurotransmitters. *TH* mutations have been reported in children with neurological disorders, including epileptic and autism spectrum disorders (Lüdecke et al., 1995; Iossifov et al., 2014; Jiao et al., 2019). However, the phenotypes of *YWHAG* mutations differ from that of *TH* mutations. Therefore, *TH* alone could not explain the pathogenicity of *YWHAG* mutations in epilepsy. Further studies are required to explore the downstream protein of *YWHAG* that are involved in epilepsy.

In conclusion, our study indicates that *YWHAG* mutations cause mild epilepsy, including childhood myoclonic epilepsy and FS. The truncating mutation identified in the big family suggests that mutations of pure haploinsufficiency are relatively less pathogenic. All *YWHAG* missense mutations were located in regions for target protein binding, suggesting a molecular sub-regional effect. Mutations with severe impairment of 14-3-3γ binding are potentially associated with severe phenotype, for which further studies are required to explore the underlying mechanism.

## Data Availability Statement

The data analyzed in this study is subject to the following licenses/restrictions: the datasets for this article are not publicly available due to concerns regarding participant/patient anonymity. Requests to access these datasets should be directed to Wei-Ping Liao, wpliao@163.net.

## Ethics Statement

Written informed consent was obtained from the individual(s), and minor(s)' legal guardian/next of kin, for the publication of any potentially identifiable images or data included in this article.

## Author Contributions

X-GY and W-PL contributed to the conception of the study. X-GY contributed to the interpretation of clinical data and drafting of the figures and the manuscript. Z-GL and JW examined the patient and participated in drafting of the manuscript. J-MD and P-XQ contributed to the collection and analysis of clinical data. P-MG helped perform the analysis with constructive discussions. W-PL provided critical review and substantially revised the manuscript. All the authors read and approved the manuscript before sending the manuscript to the journal for publication.

## Conflict of Interest

The authors declare that the research was conducted in the absence of any commercial or financial relationships that could be construed as a potential conflict of interest.
