# Supplementary material for: YWHAG Mutations Cause Childhood Myoclonic Epilepsy and Febrile Seizures: Molecular Sub-regional Effect and Mechanism
Source: Front Genet. 2021 Mar 9;12:632466. doi: 10.3389/fgene.2021.632466 (PMC7985244; doi:10.3389/fgene.2021.632466)
Supplement: Supplementary file 1 [file Table_1.DOCX]

**Supplementary Table 1 In silico analysis of *YWHAG* variants**

| **Algorithm** | **Prediction** | | | | | | | | | |
| --- | --- | --- | --- | --- | --- | --- | --- | --- | --- | --- |
|  | **c.44A>C**  **(p.Glu15Ala)** | **c.148A>C**  **(p.Lys50Gln)** | **c.169C>G (p.Arg57Gly)** | **c.169C>T**  **(p.Arg57Cys)** | **c.373A>G (****p.****Lys125Glu)** | **c.387C>G**  **(****p.Asp129Glu)** | **c.394C>T**  **(****p.Arg132Cys)** | **c.398A>C**  **(p.Tyr133Ser)** | **c.529C>A**  **(p.Leu177Ile)** | **c.398A>C**  **(p.Tyr133Ser)** |
| SIFT | Damaging | Damaging | Damaging | Damaging | Damaging | Damaging | Damaging | Damaging | Damaging | Damaging |
| Polyphen-2_HDIV | Benign | Probably_  damaging | Probably_  damaging | Probably_  damaging | Probably_  damaging | Probably_  damaging | Probably_  damaging | Probably_  damaging | Probably_  damaging | Probably_  damaging |
| Polyphen-2_HVAR | Benign | Possibly_  damaging | Possibly_  damaging | Possibly_  damaging | Probably_  damaging | Probably_  damaging | Probably_  damaging | Probably_  damaging | Probably_  damaging | Probably_  damaging |
| LRT | Deleterious | Deleterious | Deleterious | Deleterious | Deleterious | Deleterious | Neutral | Deleterious | Deleterious | Deleterious |
| MutationTaster | Disease_  causing | Disease_  causing | Disease_  causing | Disease_  causing | Disease_  causing | Disease_  causing | Disease_  causing | Disease_  causing | Disease_  causing | Disease_  causing |
| MutationAssessor | High | High | Medium | Medium | High | High | High | High | High | High |
| FATHMM | Tolerable | Tolerable | Tolerable | Tolerable | Tolerable | Tolerable | Tolerable | Tolerable | Tolerable | Tolerable |
| PROVEAN | Damaging | Damaging | Damaging | Damaging | Damaging | Damaging | Damaging | Damaging | Tolerable | Damaging |
| VEST3 | Damaging | Damaging | Damaging | Damaging | Damaging | Damaging | Damaging | Damaging | Damaging | Damaging |
| MetaSVM | Tolerable | Damaging | Damaging | Damaging | Damaging | Damaging | Damaging | Damaging | Damaging | Damaging |
| MetaLR | Tolerable | Tolerable | Damaging | Tolerable | Tolerable | Damaging | Damaging | Damaging | Damaging | Tolerable |
| M-CAP | Damaging | Damaging | Damaging | Damaging | Damaging | Damaging | Damaging | Damaging | Damaging | Damaging |
| CADD | Damaging | Damaging | Damaging | Damaging | Damaging | Damaging | Damaging | Damaging | Damaging | Damaging |
| DANN | Damaging | Damaging | Damaging | Damaging | Damaging | Damaging | Damaging | Damaging | Damaging | Damaging |
| FATHMM_MKL | Damaging | Damaging | Damaging | Damaging | Damaging | Damaging | Damaging | Damaging | Damaging | Damaging |
| Eigen | Damaging | Damaging | Damaging | Damaging | Damaging | Damaging | Damaging | Damaging | Damaging | Damaging |
| GenoCanyon | Damaging | Damaging | Damaging | Damaging | Damaging | Damaging | Damaging | Damaging | Damaging | Damaging |
| fitCons | Tolerable | Tolerable | Tolerable | Tolerable | Tolerable | Tolerable | Tolerable | Tolerable | Tolerable | Tolerable |
| REVEL | Damaging | Damaging | Damaging | Damaging | Damaging | Damaging | Damaging | Damaging | Damaging | Damaging |
| ReVe | Damaging | Damaging | Damaging | Damaging | Damaging | Damaging | Damaging | Damaging | Damaging | Damaging |
| ClinPred | pathogenic | pathogenic | pathogenic | pathogenic | pathogenic | pathogenic | pathogenic | pathogenic | pathogenic | pathogenic |
